# 5-Hydroxymethylation highlights the heterogeneity in keratinization and cell junctions in head and neck cancers

**DOI:** 10.1186/s13148-020-00965-8

**Published:** 2020-11-17

**Authors:** Siyu Liu, Marcell Costa de Medeiros, Evan M. Fernandez, Katie R. Zarins, Raymond G. Cavalcante, Tingting Qin, Gregory T. Wolf, Maria E. Figueroa, Nisha J. D’Silva, Laura S. Rozek, Maureen A. Sartor

**Affiliations:** 1 Department of Computational Medicine and Bioinformatics, University of Michigan, 100 Washtenaw Ave., Ann Arbor, MI 48109-2218 USA; 2 Department of Periodontics and Oral Medicine, University of Michigan, Ann Arbor, MI 48104 USA; 3 Department of Environmental Health Sciences, University of Michigan, Ann Arbor, MI 48109 USA; 4 Epigenomics Core, University of Michigan, Ann Arbor, MI 48109 USA; 5 Department of Otolaryngology-Head and Neck Surgery, Michigan Medicine, Ann Arbor, MI 48109 USA; 6 Department of Human Genetics and Sylvester Comprehensive Cancer Center, Miller School of Medicine, University of Miami, Miami, FL 33136 USA; 7 Department of Biostatistics, University of Michigan, Ann Arbor, MI 48109 USA

**Keywords:** Head and neck cancer, Human papillomavirus, Hydroxymethylation

## Abstract

**Background:**

Head and neck squamous cell carcinoma (HNSCC) is the sixth most prevalent cancer worldwide, with human papillomavirus (HPV)-related HNSCC rising to concerning levels. Extensive clinical, genetic and epigenetic differences exist between HPV-associated HNSCC and HPV-negative HNSCC, which is often linked to tobacco use. However, 5-hydroxymethylation (5hmC), an oxidative derivative of DNA methylation and its heterogeneity among HNSCC subtypes, has not been studied.

**Results:**

We characterized genome-wide 5hmC profiles in HNSCC by HPV status and subtype in 18 HPV(+) and 18 HPV(−) well-characterized tumors. Results showed significant genome-wide hyper-5hmC in HPV(−) tumors, with both promoter and enhancer 5hmC able to distinguish meaningful tumor subgroups. We identified specific genes whose differential expression by HPV status is driven by differential hydroxymethylation. CDKN2A (p16), used as a key biomarker for HPV status, exhibited the most extensive hyper-5hmC in HPV(+) tumors, while HPV(−) tumors showed hyper-5hmC in CDH13, TIMP2, MMP2 and other cancer-related genes. Among the previously reported two HPV(+) subtypes, IMU (stronger immune response) and KRT (more keratinization), the IMU subtype revealed hyper-5hmC and up-regulation of genes in cell migration, and hypo-5hmC with down-regulation in keratinization and cell junctions. We experimentally validated our key prediction of higher secreted and intracellular protein levels of the invasion gene MMP2 in HPV(−) oral cavity cell lines.

**Conclusion:**

Our results implicate 5hmC in driving differences in keratinization, cell junctions and other cancer-related processes among tumor subtypes. We conclude that 5hmC levels are critical for defining tumor characteristics and potentially used to define clinically meaningful cancer patient subgroups.

## Background

Head and neck squamous cell carcinoma (HNSCC) includes tumors in the oral cavity, larynx and oropharynx, and is the sixth most prevalent cancer worldwide [1, 2]. Globally, HNSCC affects approximately 680,000 patients every year, with a five-year survival rate ranging from 37 to 62% [3]. While tobacco and alcohol consumption are long-recognized risk factors, high-risk strains of human papillomavirus (HPV), in particular HPV-16, account for an increasing number of cases [4]. HPV(+) HNSCC patients generally show better therapeutic response, improved prognosis and higher overall survival [5–7]. At the molecular level, the gene CDKN2A (p16) is a marker for HPV etiology, due to its high expression level in HPV(+) tumors and common loss in HPV(−) tumors [8]. A few studies have examined molecular inter-tumor heterogeneity and identified subtypes of HNSCC [9, 10], with the characteristic differences in global gene expression profiles between HPV(+) and HPV(−) tumors and among subtypes now established. Results point to differences by HPV status and tumor subtypes in several carcinogenic pathways, including basal epithelial-to-keratinocyte proliferation, immune response, cell adhesion and induction of DNA damage that often correlate with clinical outcome.

Epigenetic differences between normal and HNSCC tumor tissue are extensive, as shown by genome-wide DNA methylation studies [11–13]. HPV(+) status is associated with hypermethylation in the promoter of several specific genes [14], and HNSCC subtypes have been identified using DNA methylation data from The Cancer Genome Atlas (TCGA) [15]. Previously, we showed that HNSCC DNA methylation profiles correlate with both patient diet and survival [14, 16] and extensive genome-wide DNA hypomethylation in HPV(−) compared to HPV(+) squamous cell carcinoma (SCC) cell lines [17], which has since been validated by others in HNSCC tumors [18].

Most of the above epigenetic data relied on bisulfite treatment of DNA, which does not distinguish between methylation (5-methylcytosine or 5mC) and hydroxymethylation (5-hydroxymethylcytosine or 5hmC). TET (ten-eleven translocation) proteins can oxidize 5mC to 5hmC and other oxidative derivatives, with 5hmC being the most abundant form in vivo [19–21]. This conversion results in a loss of transcriptional repression in promoters or enhancers, and is a common mechanism to activate genes in differentiation and development [22]. Recent studies found that 5hmC is depleted in human cancers of many different origins [23–25], yet a recent study of oral cancers found that globally elevated 5hmC is positively associated with more aggressive tumors and worse survival [26]. Genome-wide 5hmC profiles in HNSCC and in specific tumor subtypes remain uncharacterized, and virtually nothing is known regarding the association of oncogenic viruses such as HPV with 5hmC levels.

Here, we capture genome-wide hydroxymethylation profiles and examine their heterogeneity among 18 HPV(+) and 18 HPV(−) previously well-characterized HNSCC tumors. We previously characterized two distinct HPV(+) HNSCC subtypes based on gene expression and copy number variation for these 36 tumors and those from TCGA [9]. The IMU subtype is identified by a heightened immune response and more mesenchymal differentiation, whereas the KRT subtype is identified by more keratinization and viral integration events. Based on differential 5hmC profiles in other human cancers and the fundamental distinctions between HPV(+) and HPV(−) HNSCC, we reasoned that HPV infection would induce changes in hydroxymethylation, especially near differentiation and developmental genes, and corresponding genes differing by HPV status or tumor subtype. Specifically, since 5hmC levels are higher in more differentiated cells and lower in stem-like cells [27], we hypothesized an overall higher 5hmC level in HPV(−) tumors, since they tend to be more differentiated. Additionally, we predicted differential 5hmC to exist between the IMU and KRT subtypes.

We used hydroxymethylated DNA immunoprecipitation sequencing (hMeDIP-Seq) to assess 5hmC in our tumor cohort and integrated results with previously generated RNA-seq data from the same tumors [28, 29]. Results pointed to extensive differential hydroxymethylation both by HPV status and HPV(+) subtype. The 5hmC levels at both promoter and enhancer regions distinguish meaningful tumor subgroups and associate with survival. We found a strong positive correlation between hydroxymethylation and gene expression. By integrating 5hmC with gene expression, we detected important pathways enriched in comparison based on HPV status and subtypes, including keratinization and cell junctions. Finally, we found that a much higher portion of hyper-hydroxymethylated regions for HPV(−) samples fall in keratinocyte enhancer regions compared with HPV(+) samples. Since some of these enhancers can be linked to differentially expressed target genes, this result indicates that both promoter and enhancer hydroxymethylation play important roles in HNSCC gene regulation. Our results partially explain different mechanisms responsible for previously noted subtype differences and suggest that 5hmC could be a potential epigenetic target in HNSCC based on HPV status and HPV(+) subtype.

## Results

### Widespread differential hydroxymethylation between HPV(+) and HPV(−) tumors

The HNSCC cohort consisted of 18 HPV(+) and 18 HPV(−) patients, as previously determined based on RNA-seq alignment to 14 known high-risk HPV genomes. The cohort consisted of 26 males and 10 females, with an overall median age of 57 years (Additional file 1: Table S1). A total of 14 of the 18 HPV(+) patients were infected by subtype HPV16, and most were former or current smokers. hMeDIP-Seq was performed on these 36 HNSCC tumors to define their genome-wide hydroxymethylation signatures, examine how they differed by HPV status and tumor subtypes, and assess their relationship with clinical variables.

All 36 samples resulted in sufficient quality of data and hundreds of thousands of identified 5hmC peaks, with the sequencing depth ranging from 67.7 to 152.3 million reads mapped. The number of peaks detected generally ranged from 208,000 to 480,000. As expected, the number of peaks was positively correlated with the total number of reads mapped. There were no significant differences between HPV(+) and HPV(−) tumors based on peak numbers (*p* value = 0.355, Wilcoxon signed-rank test, Additional file 1: Figure S1A), even after accounting for millions of reads mapped (*p* value = 0.287, ANOVA test).

**Fig. 1:**
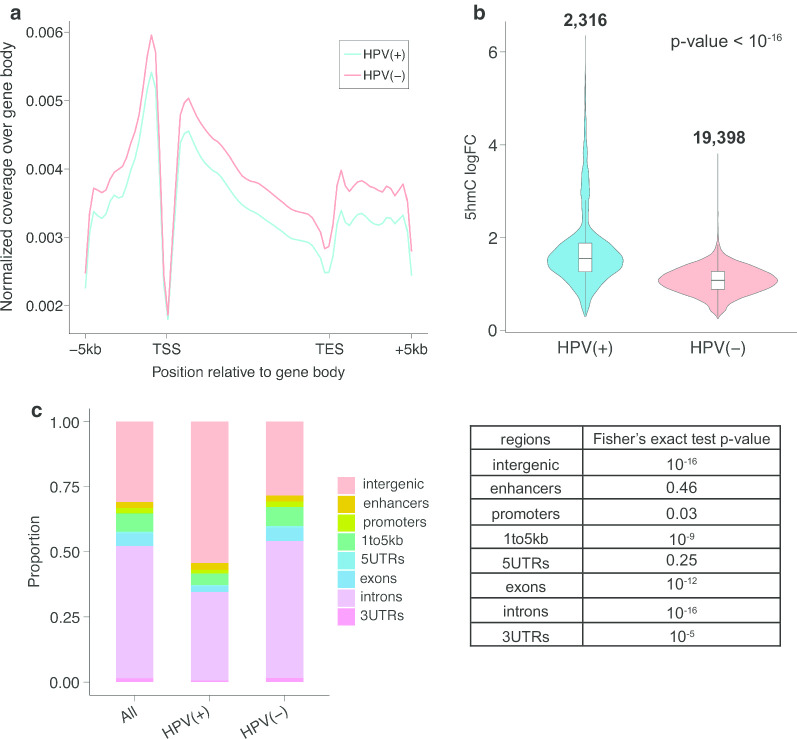
The level and distribution of hydroxymethylation varies by HPV status. **a** Global 5hmC distribution pattern over gene bodies in both HPV(+) and HPV(−) samples. **b** Violin plot of 5hmC logFC in HPV(+) tumors (left) and HPV(−) tumors (right). The number on top indicates the total number of peaks being tested. Wilcoxon signed-rank test *p* value < 10^–16^. **c** The distribution of hyper-5hmC peaks from HPV(+) and HPV(−) samples, where first column represents the combination of HPV(+) and HPV(−) tumors. The table on the right displayed the *p* values from Fisher’s exact test between HPV(+) and HPV(−) HNSCC, where exons and introns showed a *p* value of 10^–12^ and 10^–16^ respectively

In general, a much higher level of hydroxymethylation (hyper-5hmC) was reported in HPV(−) HNSCC. Enrichment of 5hmC levels was plotted over gene bodies, and we observed a consistently higher level of 5hmC in gene bodies across the genome in HPV(−) tumors (Fig. 1a). Consistent with previous studies, the average gene body profiles revealed a dip around the transcription start site (TSS) and transcription end site (TES) regions [30].

A total of 19,398 differentially hydroxymethylated regions (DhMRs) were detected as hyper-5hmC in HPV(−), as opposed to only 2316 in HPV(+) tumors (*p* value < 10^–5^). Most differential peak widths were narrow, between 100 and 200 bp, and HPV(−) DhMRs were slightly longer than those for HPV(+) (Additional file 1: Figure S1B). Although fewer peaks were hyper-5hmC in HPV(+) samples, they were in general stronger than the HPV(−) DhMRs, with larger fold change (Fig. 1b).

Of the 2316 DhMRs in HPV(+) tumors, about 46% were annotated to genes, with the majority in introns. Of the 19,398 DhMRs in HPV(−) tumors, more than 72% were annotated to genes, also with the majority in introns. By comparing the distribution of HPV(+) and HPV(−) DhMR annotations to the annotations of random genomic regions, we found a significantly higher proportion of DhMRs were in exons (5.34%) and introns (52.77%) in HPV(−) samples, as opposed to a smaller percentage in exons (2.32%) and introns (33.98%) for HPV(+) DhMRs (Fig. 1c). Together, these results suggest that HPV positivity in HNSCC is linked to a reduced hydroxymethylation signature both in and around genes.

### Genes and pathways with hyper-5hmC in HPV(+) tumors

A total of 623 genes were associated with hyper-hydroxymethylation in HPV(+) tumors. CDKN2A (p16), the most important biomarker of HPV status in clinical tests [31], had one of the highest number and also the most significant HPV(+) DhMRs. In total, 35 genes had hyper-5hmC in promoter regions of HPV(+) tumors. As a prime example, 5hmC was enriched at the CDKN2A promoter in HPV(+) cases compared to HPV(−), a difference that was independent of copy number variations since this is controlled for by the use of input references. This is important, since loss of the CDKN2A locus is known to occur in HPV(−) cases. The raw coverage depth by sample and peak signal values of CDKN2A both showed great deviation in the promoter and along the gene body between HPV(+) and HPV(−) tumors (Additional file 1: Figure S2A). Other important genes found with HPV(+) DhMRs are listed in Additional file 1: Table S2A.

Pathways enriched with higher 5hmC in HPV(+) HNSCC included desmosome, activation of NF-kappaB-inducing kinase activity, oxidoreductase activity and mesenchymal cell differentiation (FDR < 0.1) (Additional file 1: Figure S3). Mesenchymal development associated with epithelial to mesenchymal transition (EMT) is consistent with the higher risk of distant metastasis in HPV+ HNSCC. A total of 27 genes displayed higher 5hmC in HPV(+) tumors in mesenchymal cell differentiation and development, including the key EMT genes SNAI2, BMP2, SMAD2 and TGFB2, which are part of the TGFβ / Bone Morphogenic Protein (BMP) signaling pathway [32, 33].

### Genes and pathways with hyper-5hmC in HPV(−) tumors

A larger number of genes, 5584, were found to be hyper-hydroxymethylated in HPV(−) HNSCC, of which 372 genes contained at least one promoter region DhMR. Some of the most important genes with promoter DhMRs were BCAR1, which plays crucial roles in metastasis and cell adhesion, and MMP2, which functions in EMT and immune response in multiple cancer types. We found 204 genes to harbor more than 10 HPV(−) hyper-5hmC regions. CDH13, a gene encoding a member of the cadherin superfamily that functions in cell-to-cell adhesion and is involved in several diseases, had 83 DhMRs. The peak signal values over each DhMR indeed demonstrated a higher level of hydroxymethylation in HPV(−) compared with HPV(+) (Additional file 1: Figure S2B). A more detailed list of important genes with HPV(−) DhMRs can be found in Additional file 1: Table S2B.

Pathway enrichment results identified cell morphogenesis, cell death, cell motility and cytoskeletal rearrangement/cell–cell junction being among the significantly enriched (FDR < 0.1) (Additional file 1: Figure S3). Several previously verified HNSCC-related genes were in a top enriched pathway. For example, frequently mutated HNSCC genes ERBB2, FGD1, NOTCH1, NR4A2, SEMA3E and ARAP3 had HPV(−) DhMRs in cell morphogenesis, and other head and neck-relevant genes such as CTGF, PKN2, TERT, TGFBR2 and TP63 in signal transduction had DhMRs.

### Main sources of heterogeneity in hydroxymethylation in promoter and enhancer regions

**Fig. 2:**
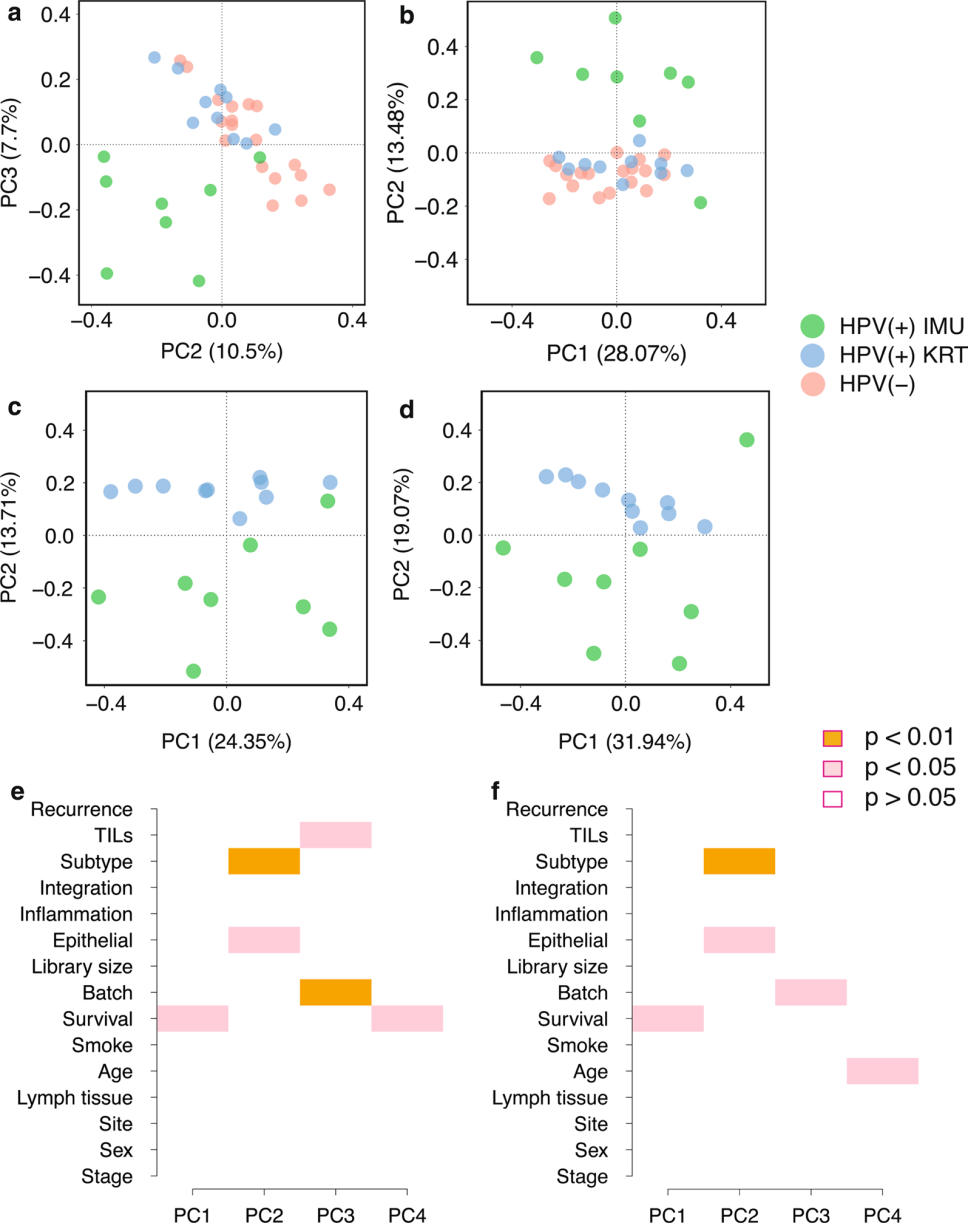
Principle component analysis illustrated sources of heterogeneity in 5hmC levels among HPV(+) HNSCC tumors. **a** PC2 vs PC3 for proximal promoter regions. **b** PC1 vs PC2 for custom defined enhancers regions showed clear separation between the IMU subtype and the rest on PC2, which contributed 10.5% and 13.48% of the total variance respectively. **c**, **d** PC1 versus PC2 for both proximal promoters and custom defined enhancers showed clear separation by subtype within HPV(+) tumors. **e**, **f** The SVD analysis on proximal promoters and enhancers demonstrated several relevant clinical variables, such as survival, percentage of epithelial tissue and subtype, which significantly correlated with each principle component in 18 HPV(+) samples

We next sought to understand the sources of 5hmC heterogeneity in our cohort using principle component analysis (PCA). Interestingly, the greatest source of heterogeneity in promoter 5hmC profiles did not distinguish HPV(+) from HPV(−) tumors, but rather one subtype of the HPV(+) tumors (IMU) from all other tumors (see PC2 in Fig. 2a, c). Consistent with the findings in Zhang, et al. [9], the KRT subtype groups closer with HPV(−) HNSCC, partially due to the shared similarities of heightened keratinization. We thus sought to determine which, if any, known variables could explain the top PCs among HPV(+) tumors. Correlations between the top principle components and clinical, demographic and batch information were calculated using singular value decomposition (SVD) analysis on proximal promoter regions for the 18 HPV(+) samples. PC1 was correlated with survival (*p* value < 0.05), while both subtype (*p* value < 0.01) and percentage of epithelial tissue (*p* value < 0.05) were significant in PC2 (Fig. 2e). TILs (tumor-infiltrating lymphocytes) score and batch effect were correlated with PC3, while survival and recurrence (*p* value < 0.01) were correlated with PC4.

In terms of 5hmC heterogeneity in enhancer regions among the 36 tumors, a similar distinction between IMU and the other tumor samples was observed (see PC2 in Fig. 2b, d). Correlating variables with the top enhancer PCs for the 18 HPV(+) samples, survival was again a significant factor in PC1 (*p* value < 0.05), and subtype (*p* value < 0.01) and epithelial tissue (*p* value < 0.05) were again both significant in PC2 (Fig. 2f). However, the separation between the HPV(+) IMU subtype and the rest was not observed for 5hmC gene body levels (Additional file 1: Figure S4A-D). Instead, SVD analysis on gene body 5hmC showed other relevant clinical features associated with the top PCs, including survival, lymphocyte tissue and HPV integration status (Additional file 1: Figure S4E-F).

### Differential hydroxymethylation and enriched pathways between the IMU and KRT tumor subtypes

Compared with the IMU subtype, an overall higher level of 5hmC was observed in the KRT subtype across the gene bodies, which was closer to the 5hmC levels of HPV(−) tumors (Additional file 1: Figure S5A).

In terms of differential 5hmC between the two HPV(+) subtypes, there were significantly more instances of hyper-hydroxymethylation in the IMU subtype samples. A total of 63,859 hyper-5hmC regions were found in the IMU subtype, compared with only 1833 hyper-5hmC regions in the KRT subtype. Only 838 (1.3%) of these regions were also among those found different by HPV status, out of which the majority (771 peaks) were hyperhydroxymethylated in HPV(+) and IMU tumors (Additional file 1: Figure S5B). Similar to the annotation of DhMRs based on HPV status, the majority of both IMU and KRT DhMRs were mapped to introns (Additional file 1: Figure S5C). A detailed list of important genes with hyper-5hmC in HPV(+) IMU or KRT tumors can be found in Additional file 1: Table S3A and S3B, respectively. Interestingly, cancer genes CDH13 and BCAR1 were found with multiple KRT DhMRs, and they were also important genes for hyper-5hmC in HPV(−) tumors, which is consistent with the previous finding that the KRT subtype shares more similarities with HPV(−) HNSCC.

The top enriched pathways marked by hyper-5hmC in the IMU subtype include cornification, epidermis development, keratinocyte differentiation, keratinization and cell differentiation (Additional file 1: Figure S6). For the KRT subtype, cornification and keratinocyte differentiation also appear in the top enriched pathways. However, multiple pathways relevant to cytoskeleton organization or cell–cell junction, including cell adhesion and cytoskeleton structure, were found only in the KRT subtype and were within the top 20 enriched terms (Additional file 1: Figure S6). These terms were also significantly hyper-hydroxymethylated in HPV(−) compared to HPV(+), further explaining the similarity of the HPV(+) KRT subtype to HPV(−) HNSCC.

### Hydroxymethylation is highly associated with gene expression in HNSCCs

**Fig. 3:**
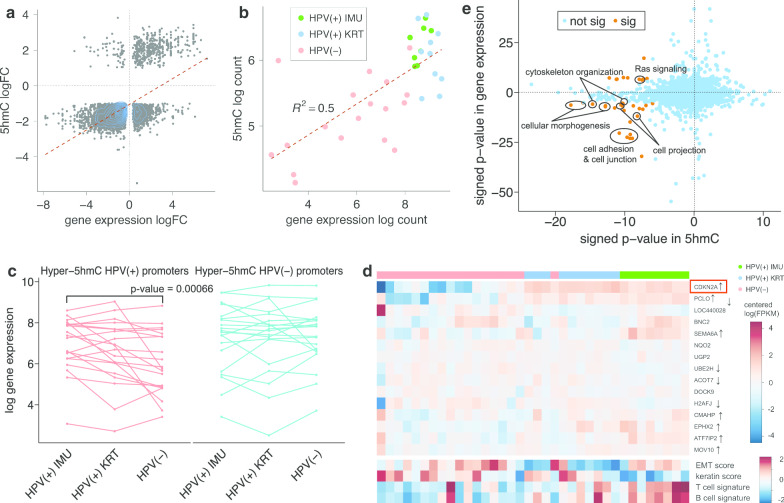
5hmC in HNSCC is highly correlated with gene expression. **a** Scatterplot showing the positive correlation between gene expression and hydroxymethylation in HPV(+) and HPV(−) samples (Pearson correlation coefficient = 0.62). The top half represents genes that are significantly up-regulated in HPV(+) tumors, and the right half represents genes that are significantly hyper-hydroxymethylated in HPV(+) tumors. **b** Scatterplot showing a strong correlation between log gene expression and log 5hmC of 5 kb intron region (chr9:21975000–21980000) of CDKN2A. HPV(+) samples were concentrated near the top right corner, indicating that both their gene expression and 5hmC coverage were higher compared with HPV(−) samples. **c** Spaghetti plot of log gene expression for top 20 genes with at least one DhMR in their promoter region for HPV(+) IMU, HPV(+) KRT and HPV(−) samples, respectively. **d** Heatmap showing the expression levels of sufficiently expressed genes with at least one HPV(+) DhMR in their promoter, which were mostly well clustered based on HPV status (marked with black and grey at the top). Most genes were significantly higher expressed in HPV(+) samples, such as CDKN2A. ↑ indicates genes that are also up-regulated in HPV(+) HNSCC, and ↓ indicates up-regulation in HPV(−) tumors. Keratin and EMT scores are measurements of keratinization level and EMT level, and T cell signature and B cell signature represent degree of immune response. Generally there was a higher level of keratinization and EMT in HPV(−) samples, while the immune response is more significant in HPV(+) samples. The detailed calculation can be found in Zhang et al. [9]. **e** Enrichment analysis results for gene expression vs hydroxymethylation by HPV status. Each dot represents one GO term, and the color denotes the significance (yellow: significant; blue: not significant). Signed *p* values are defined as > 0 to indicate up-regulation in HPV(+) samples or hyper-hydroxymethylation in HPV(+) samples, and < 0 to indicate upregulation in HPV(−) samples or hyper-hydroxymethylation in HPV(−) samples

RNA-seq data on the same 36 HNSCC samples were previously analyzed, resulting in 1887 up-regulated and 1644 down-regulated genes in HPV(+) samples (FDR < 0.05 and absolute fold change > 2) [9, 34]. A clear pattern of association can be observed between gene expression and 5hmC (Pearson’s correlation coefficient = 0.62; odds ratio (OR) = 64.5) (Fig. 3a), suggesting that 5hmC likely drives many of these observed differences. This positive correlation still holds when comparing gene expression with 5hmC logFC at enhancer, promoter and gene body separately, with gene body regions showing the strongest correlation (Pearson’s correlation coefficient = 0.53, Additional file 1: Figure S7). The majority of genes (61.4%) are upregulated and hyper-hydroxymethylated in HPV(−) tumors, such as cell adhesion genes (including CDH13, CDH11, CDH2, CD44, GLI2, COL4A6), immune response genes (including TGFBR2, CD109, BCAR2, TIMP2, MMP2) and keratinization genes (CDH13, CD109, CDR, PALLD). In particular, TIMP2 and MMP2 also function in tumor invasion.

For CDKN2A, we determined that differential 5hmC was especially prominent in a 5 kb region 5′ of the second exon (chr9:21975000–21980000) (Additional file 1: Figure S2A). To assess the association of this particular region with CDKN2A expression, we calculated 5hmC coverage per sample and found that 5hmC at this single region explained half of the variability in CDKN2A gene expression levels among the samples (Pearson’s correlation coefficient = 0.7). (Fig. 3b).

We next sought to determine the extent to which hydroxymethylation in promoter regions explained differences in gene expression. Twenty genes had at least one hyper-5hmC region in HPV(+) compared to HPV(−) in their promoter region, after excluding very low expressed genes. The expression of these genes was indeed significantly higher in HPV(+) IMU than HPV(−) samples (ANOVA *p* value = 0.00066) (Fig. 3c). The distinction between HPV(+) and HPV(−) samples was particularly clear for CDKN2A (Fig. 3d). On the other hand, 296 genes had at least one hyper-5hmC region in HPV(−) in the promoter. However, there was no significant distinction found in the expression of the top 20 of these genes in any of the three comparisons (Fig. 3c), leading us to hypothesize that the upregulation of genes due to hyper-5hmC in HPV(−) tumors is due to differences at enhancers rather than promoters. By using the genes with hyper-5hmC peaks on the promoter regions, we built networks using the shortest paths. The result showed that p16INK4 (CDKN2A) and p14ARF (alternate reading frame protein product of the CDKN2A locus) are the two center nodes for HPV(+) samples (Additional file 1: Figure S8A), while SMAD3, ABCC2 and IL32 are center nodes in the HPV(−) network (Additional file 1: Figure S8B).

Overall, we identified 35 GO terms both enriched with differential hydroxymethylation (whether proximal to the promoter or distal elements) and differential expression between HPV(+) and HPV(−) samples (Fig. 3e). Twenty-five of these 35 GO terms were upregulated and hyper-hydroxymethylated in HPV(−) samples, including adherens junction, cell morphogenesis, chemotaxis and Ras signaling (Additional file 1: Table S4). This finding suggests that the higher expression of cell junction genes in HPV(−) tumors is at least partly regulated by hydroxymethylation. It also suggests that HPV infection could impact many cell junction biomarkers via the active demethylation process.

Next, we explored the enriched pathways based on HPV(+) subtype, finding 38 GO terms with significant hyper-5hmC and up-regulation in the IMU subtype, including cell migration, phosphorylation, MAPK cascade and cytokine-mediated signaling pathway (Additional file 1: Figure S5D, Table S5A). Conversely, there were 11 GO terms with significant hyper-5hmC and up-regulation in the KRT subtype, including cell–cell junction, keratinization and epidermal cell differentiation, which is consistent with the more differentiated nature of the KRT subtype (Additional file 1: Table S5B).

### HPV(−) tumors are more hydroxymethylated in keratinocyte enhancer regions than HPV(+) tumors

**Fig. 4:**
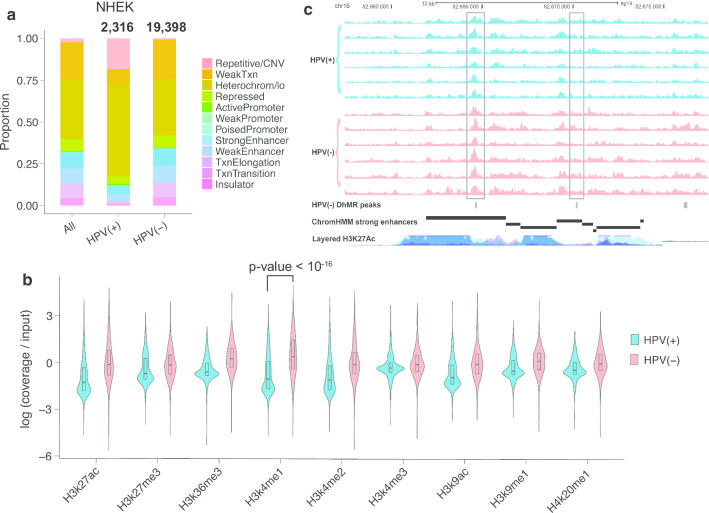
HPV(−) tumors have strong hyper-5hmC regions in epithelial and keratinocyte enhancer regions. **a** HPV(−) HNSCCs showed much higher portion of DhMRs in strong and weak enhancers than both random regions and HPV(+) tumors. Regions were defined using the ChromHMM track for NHEK cells. The number on top indicates the total number of peaks being tested. **b** Violin plot showing ChIP-seq log(coverage/input) values for top 1000 HPV(+) and top 1000 HPV(−) hyper-5hmC peaks for 9 histone marks. Wilcoxon signed-rank test showed a *p* value < 10^–16^ for H3k4me1 peaks. **c** UCSC Genome Browser view of NHEK strong enhancers near CDH13. Data shown are 5hmC profiles for 6 representative HPV(+) samples (upper 6 tracks) and 6 representative HPV(−) samples (lower 6 tracks), showing 3 regions of high 5hmC level for HPV(−)

Since 5hmC has been shown to be an important mark in enhancer regions [35], we revealed the distribution of hydroxymethylation across samples in relation to different chromatin states, including enhancers, in primary Normal Human Epidermal Keratinocyte (NHEK) cells. NHEK cells are isolated from the epidermis of juvenile foreskin or adult skin and are similar to head and neck tissue both morphologically and physiologically [36]. Around the center of NHEK active enhancers and weak enhancers, we observed much higher levels of 5hmC in HPV(−) tumors compared with both HPV(+) subtypes, although all of them showed the expected pattern of an increase around the enhancer centers (Additional file 1: Figure S9). Enhancers also had more differences in hydroxymethylation than promoter regions, as indicated by strikingly more DhMRs in strong enhancers than in active promoters for both HPV(+) and HPV(−) tumors (Fig. 4a, Table 1). Fisher’s exact test showed an odds ratio (OR) of 2.56 (*p* value < 10^–16^) for strong enhancers and 2.43 (*p* value < 10^–16^) for weak enhancers comparing HPV(+) vs HPV(−) samples. This disparity between HPV(+) and HPV(−) samples is consistent with the fact that HPV(−) HNSCCs tend to be more differentiated than HPV(+) HNSCC.

**Table 1:** Number and percentage of HPV(+), HPV(−), HPV(+) IMU and HPV(+) KRT DhMRs that overlap with keratinocyte enhancers, promoters and super-enhancers

	Strong enhancers	Weak enhancers	Active promoters	Weak/poised promoters	Super-enhancers
HPV(+) DhMRs (2316)	94; 4.06%	110; 4.75%	17; 0.73%	29; 1.25%	34; 1.47%
HPV(−) DhMRs (19,398)	1909; 9.84%	2136; 11.01%	66; 0.34%	273; 1.41%	716; 3.45%
HPV(+) IMU DhMRs (63,859)	4971; 7.65%	6578; 10.30%	225; 0.35%	777; 1.22%	1374; 2.15%
HPV(+) KRT DhMRs (1833)	230; 12.66%	197; 10.75%	8; 0.44%	20; 1.09%	117; 6.38%

We reconfirmed the higher keratinocyte (NHEK) enhancer 5hmC levels in HPV(−) tumors using ChIP-seq data for the histone mark H3K4me1, a mark for active and primed enhancers. Visualizing the H3K4me1 signals for the top 1000 hyper-5hmC regions for HPV(+) and HPV(−) tumors separately showed the highest signal value within HPV(−) hyper-5hmC regions (Fig. 4b). Similar to previous findings, this trend was not observed for H3K4me1 signals in HPV(+) tumors, indicating that HPV(−) samples have higher levels of 5hmC in keratinocyte enhancer regions.

The target genes of strong enhancers with at least one DhMR were determined using publicly available ChIA-PET data (see Additional file 1: Supplementary Methods). In order to study the impact of enhancer hydroxymethylation on target gene expression, we specifically focused on target genes that were also differentially expressed. There were 5 and 66 hyper-hydroxymethylated enhancers associated with differentially up-regulated genes in HPV(+) and HPV(−) tumors, respectively. In particular, CLDN1, a cell-to-cell adhesion gene, was the target gene of a HPV(+) enhancer DhMR and was also up-regulated in HPV(+) tumors. Conversely, differentially expressed genes CDH13, BCAR1 and TIMP3 not only displayed HPV(−) enhancer hyper-5hmC, but also contained multiple HPV(−) DhMRs in their exonic and intronic regions. Multiple strong enhancers showed a higher level of hydroxymethylation in HPV(−) samples in all three of these genes (Fig. 4c).

### Expression of invasion gene MMP2 in HPV(+) and HPV(−) cell lines

Our results show that many immune response genes, such as BCAR1, TIMP2 and MMP2, were both higher expressed and hyper-hydroxymethylated in the promoter and sometimes enhancer regions in HPV(−) tumors. In a previous study, 5hmC was shown to be positively correlated with depth of tumor invasion in colorectal cancer [37], and depletion of TET1, which could oxidize 5mC to 5hmC, could facilitate cell invasion [38]. Along these same lines, multiple studies reported that high levels of the MMP2 protein were linked with larger tumor size and more tumor invasion [39]. Therefore, we hypothesized that hyper-hydroxymethylation and higher mRNA levels of MMP2 would result in higher MMP2 secreted protein, which can lead to stronger invasion in HPV(−) HNSCC.

**Fig. 5:**
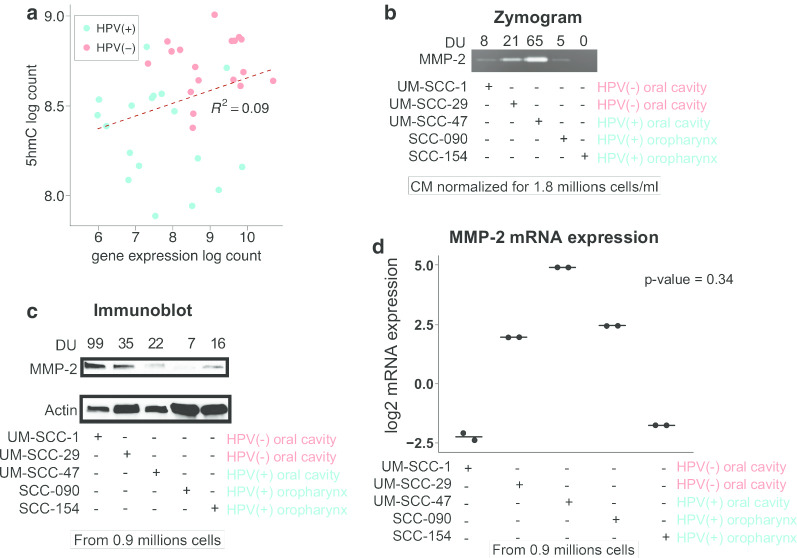
Protein expression level of MMP2 in 2 HPV(−) and 3 HPV(+) HNSCC cell lines. **a** Scatterplot showing a correlation between log gene expression and log 5hmC of MMP2 gene. HPV(−) samples were concentrated near the top right corner, indicating that both their gene expression and 5hmC coverage were higher compared with HPV(+) samples. **b** Zymogram and **c** immunoblot indicated the secreted and intracellular level of MMP2 protein respectively. **d** Dot plot showing the mRNA level of MMP2 with two replicates. After removing the HPV(+) oral cavity cell line, which is an outlier, Wilcoxon signed-rank test showed a *p* value = 0.34

First we reconfirmed that MMP2 in HPV(−) tumors showed both higher gene expression and overall higher 5hmC compared with HPV(+) tumors, and there is a positive correlation between the gene expression and 5hmC (Fig. 5a). Next, we assessed the secreted and intracellular protein levels of MMP2 in two HPV(+) oropharynx, one HPV(+) oral cavity and two HPV(−) oral cavity cell lines. The zymogram results showed higher levels of secreted MMP2 in HPV(−) cells compared to HPV(+) oropharynx cell lines, but not HPV(+) oral cavity (Fig. 5b). Intracellular MMP2 was also higher in the HPV(−) cell lines than the HPV(+) oropharynx cells (Fig. 5c). There is no clear distinction in the MMP2 mRNA levels between HPV(−) oropharynx and HPV(+) oral cavity cell lines (Fig. 5d).

## Discussion

5-Hydroxymethylcytosine has been shown to be depleted in various human cancers and is known to be more concentrated in differentiated cells [23, 27]. Stem cells, which are closest to the basal epithelial cells in HNSCCs, are known to have lower 5hmC levels, especially in the gene and enhancer regions required for differentiation [28, 30]. For our purposes, those differentiation-specific regions would be epithelial and keratinocyte-specific genic and enhancer regions. Differences between HPV(+) and HPV(−) HNSCC are extensive in terms of prognosis, tumor recurrence patterns and survival [5, 7, 8]. Similarly, molecular studies have shown marked differences in gene expression, DNA copy numbers and DNA methylation profiles by HPV(+) status [9, 15, 17]. For instance, multiple studies showed genome-wide DNA hypomethylation in HPV(−) HNSCC tumors [18], and the differential methylation of certain tumor suppressor genes could be potential markers for early HNSCC diagnosis [12].

The HPV lifecycle is tightly linked to epithelial cell differentiation, with HPV initially infecting the undifferentiated basal epithelial cells, and concluding its life cycle in differentiated keratinocytes. Upon HPV oncogene integration, heightened keratinization often occurs, potentially affecting metastatic risk [40]. Our group has shown that patients with integrated HPV E6 & E7 had significantly worse overall survival [41]. Unlike most cancers, evidence does not suggest that less differentiated HNSCCs are associated with worse survival; indeed, studies have suggested that more differentiated keratinocytes are associated with worse survival in oropharyngeal cancer [42]. Due to the limited sample size of this study, we did not observe any significant survival difference between the two HPV(+) subtypes, where 2 and 0 deaths were reported after 36 months follow-up in the KRT and IMU subtype, respectively. Another recent publication on meta-analysis of HPV(+) OPSCC followed the observed trend of the IMU subtype having the best survival (labeled Cl1 in the paper) and the subtype with 100% HPV integration (similar to our KRT subtype; Cl2 in paper) having worse survival [43].

**Fig. 6:**
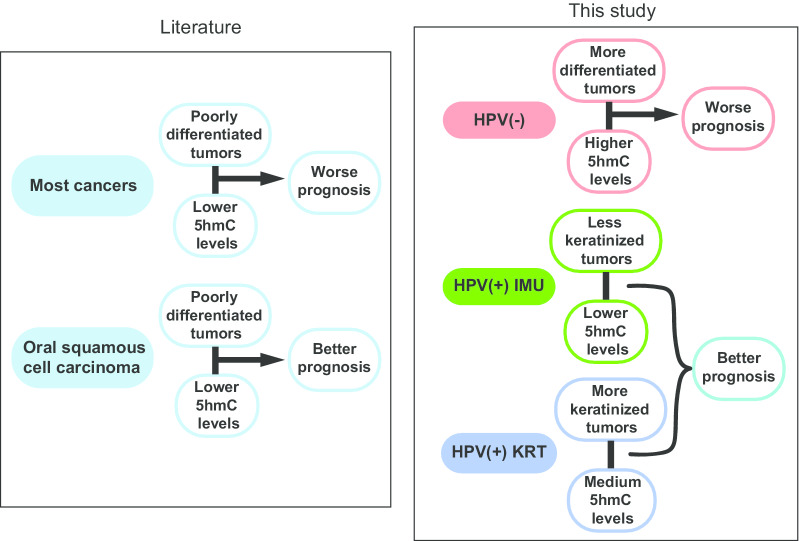
Schematic chart summarizing the correlation between 5hmC level and prognosis within different tumor types in published literature (left) and our study (right)

Although 5hmC tends to be overall lower in cancers, others have observed lower 5hmC in oral cancers to actually be indicative of better prognosis [26] (Fig. 6). This could be due to the confounding effect of HPV; in this study, we found lower 5hmC in HPV(+) patients, who have less differentiated tumors and better prognosis (Fig. 6). However, mesenchymal differentiation may also lead to loco-regional or distant metastasis; thus, the complete relationship between differentiation and metastasis appears to be complex, as suggested by studies in oral cancer [44]. Among our differential 5hmC genes, only CDK6 was identified as a clinically actionable target. CDK6 was hyper-hydroxymethylated in HPV(−) tumors at seven intronic regions, is targeted in the treatment of certain breast cancers [45] and recently showed response in the treatment of oral squamous cell carcinoma [46]. CD20, which is regulated by the epigenetic markers NFκB and SMAD2/3 [47], is targeted in chronic lymphocytic leukemia and follicular lymphoma [48]. The NFκB pathway and SMAD2/3 genes were identified as important 5hmC markers in our study, suggesting a potential use of B cell markers in the immunotherapy of HNSCC.

Our study is the first to characterize genome-wide DNA hydroxymethylation in head and neck cancers. Among differentiation genes, we found strong hyper-5hmC in HPV(−) tumors especially concentrated in cell junction and cell adhesion pathways, which is consistent with previous findings that HPV(−) HNSCC is more differentiated compared with HPV(+) HNSCC. The overall higher level of hydroxymethylation in HPV(−) HNSCCs is also consistent with observed overall higher levels of DNA methylation in HPV(+) oropharyngeal cancer cases, considering the antagonizing effect of methylation and hydroxymethylation [18]. Similar to DNA methylation, profiles of hydroxymethylation were highly affected by HPV status, particularly for p16. The great majority of hyper-hydroxymethylated genes were in HPV(−) HNSCC, many of which play important roles in cancer pathways. CDH13, a cell–cell adhesion gene and a member of the cadherin superfamily, was upregulated in HPV(−) tumors and also had the highest number of hyper-5hmC regions in HPV(−). Interestingly, other major cadherin family genes (e.g., CDH1 and CDH11) are hypermethylated in HPV(+) HNSCC [17, 49]. Some genes known to have differential methylation by HPV status also displayed differential 5hmC in the opposite direction, including cell adhesion genes COL4A6 and BCAR1, and tumor suppressor genes TIMP3 and SFRP4.

Even more than distinguishing HNSCC tumors by HPV status, 5hmC profiles distinguished the IMU HPV(+) subtype from the KRT HPV(+) subtype and HPV(−) tumors. The KRT subtype is more similar to HPV(−) HNSCC than the IMU subtype based on gene expression [9]. Consistent with this, we found the same based on 5hmC, with a much higher level of 5hmC found in the KRT subtype, which is furthermore consistent with the more differentiated nature of this subtype. We also found cancer pathways such as cell migration enriched with hyper-5hmC in the IMU subtype, while cornification and keratinization were significantly enriched with hyper-5hmC in the KRT subtype.

A recent study on hydroxymethylation of pancreatic cancer showed positive correlation between 5hmC and open chromatin generated ATAC-seq in both cancer and control cells [50]. While we did not have ATAC-seq data available, in our study we found a similar correlation with ChromHMM tracks of NHEK cells [51], and the especially strong enrichment of 5hmC on enhancers in HPV(−) HNSCC could partially be attributed to its more differentiated and/or malignant nature.

In summary, our comprehensive characterization of the genome-wide hydroxymethylation profiles in HNSCC revealed significant differential hydroxymethylation both by HPV status and between HPV(+) subtypes. We report the significance of CDKN2A hydroxymethylation by HPV status, as well as many other cancer-related genes, such as CDH1, TIMP3 and SFRP4. Overall, the results are closely in line with current knowledge of differences by HPV status, including differences in DNA methylation. We also discovered the important role of the less reported gene CDH13 in HNSCC, and that the differential hydroxymethylation was especially concentrated in CDH13 enhancer regions.

## Conclusions

In conclusion, although 5hmC marks genes across a wide array of cellular processes, 5hmC profiles highlight genes turned on during differentiation, and can therefore be used for an in-depth characterization of the differentiation state of tumors. Thus, genome-wide 5hmC analysis is beneficial, especially to the extent that differentiation state affects carcinogenic pathways, including cell junctions and adhesion, invasion and migration in our study. Our data suggest that 5hmC profile for the IMU subtype could be a useful biomarker in HPV positive cancers and should be explored further.

As the first study to characterize the genome-wide 5hmC profile in HNSCC, we identified significant genome-wide hyper-5hmC in HPV(−) tumors, with both promoter and enhancer 5hmC levels being clinically relevant and able to distinguish meaningful tumor subgroups. We also implicated 5hmC in key cancer-related processes that determine the likelihood of metastasis in head and neck cancer. genes. Clinically, therapeutic de-escalation schedules are being introduced for HPV(+) patients, but the current challenge to such changes includes better identification of the small subset of HPV+ cancer patients that have poor prognosis. Our study has important implications that 5hmC levels are crucial in defining tumor characteristics and potentially used to define clinically meaningful cancer patient subgroups for many cancer types.

## Methods

### Patient recruitment and hMeDIP-seq protocol

From 2011 to 2013, we identified 36 incident HNSCC patients with pre-treatment oropharynx or oral cavity squamous cell carcinoma at Michigan Medicine Hospital. HPV status was determined based on p16 staining and RNA-seq, as previously described [9, 41]. The details of tumor tissue acquisition can be found in Additional file 1: Supplementary Methods. After DNA extraction, the quality of the 36 DNA samples was measured by TapeStation genomic DNA kit (Agilent, Santa Clara, CA), followed by quantitation assessment by Qubit broad range dsDNA (ThermoFisher, Carlsbad, CA). Enzymes, PCR primers and indexed adaptors were supplied by New England BioLabs (Ipswich, MA) and Integrated DNA Technologies (Coralville, CA), respectively.

A total of 1 μg of genomic DNA was used for shearing, blunt-end repair and phosphorylation process, and a single adenine nucleotide was then added to the 3′ end of the resulting fragments for ligation preparation. DNA was cleaned by Qiagen’s MinElute PCR purification columns (Qiagen, Germantown, MD). DNA samples were denatured and resuspended in ice-cold immunoprecipitation buffer after the addition of DNA spike-ins for hMeDIP (Diagenode Denville, NJ). At this stage, 10% volume of the DNA solutions were kept as inputs, and immunoprecipitation overnight at 4 °C with rotation was performed on the remaining solution, after adding a 5hmC-specific antibody (Cat # 39791, Active Motif, Carlsbad, CA). The 5hmC-enriched DNA fragments (IP) were released from the antibody and cleaned-up by Proteinase K (ThermoFisher, Carlsbad, CA) and AMPure XP beads (Beckman Coulter, Brea, CA), respectively. In order to evaluate the percent enrichment over input in the IP, qPCR with primers for spike-ins was conducted. For samples with good percent enrichment over input, PCR amplification was performed for library production, followed by cleaning with AMPure XP beads and quantification with the Qubit assay (ThermoFisher, Carlsbad, CA) and TapeStation High Sensitivity D1000 kit (Agilent, Santa Clara, CA). Each hMeDIP-seq sample with paired input was sequenced on a single lane of an Illumina HiSeq 2500, generating single-end, 50 bp reads.

### hMeDIP-seq data analysis and peak finding

The main analysis steps were conducted using the Methylation Integration (*mint*) pipeline [52]. Sample quality was first assessed with FastQC [53], and then, reads were aligned with bowtie2 [54] after adapter and quality trimming with Trim Galore! Peaks for each sample compared to input, i.e. the genome-wide regions of hydroxymethylation, were identified using MACS2.

Differential peaks, i.e. differentially hydroxymethylated regions (DhMRs) between HPV(+) and HPV(−) samples, or between HPV(+) subtypes, were identified using PePr [55]. PePr takes replicates into account using a negative binomial model while improving variability estimates using information from neighboring sites. Differential peaks were called with false discovery rate (FDR) < 0.05 and fold change (FC) > 2. Peaks were annotated using the R Bioconductor package *annotatr* [56]. Peaks annotated to X and Y chromosomes were excluded to avoid confounding by sex. The overall 5hmC levels over gene bodies were calculated using MACS2 peaks with *metaGeneProfile* function in HOMER [57].

### Principle component analysis (PCA) and singular value decomposition (SVD) analysis

PCA was performed using *prcomp* function in R, with the use of hMeDIP-seq counts in proximal promoters, gene bodies and custom enhancer regions. X and Y chromosome reads were removed to avoid sex bias. The *bedtools intersect* function was used to obtain 5hmC counts in promoter regions (1 kb before to 1 kb after TSS’s) and gene bodies (from TSS to TES), followed by normalization by manual specification of library sizes in *DESeq2*, with the input values as covariate. The background was taken into account by calculating the log2 fold change for each region. SVD analysis was performed on the top principal components using the Bioconductor package *ChAMP* [58], to study correlation with variables of interest.

### Additional analyses

Detailed analysis methods for (1) generation of custom enhancer definitions; (2) RNA-seq analyses and association with 5hmC; (3) gene set enrichment testing on hMeDIP-seq and RNA-seq data; (4) keratinocyte enhancer regions download and analysis; (5) experimental validation with HPV(+) and HPV(−) cell lines are available in the Additional file 1: Supplementary Methods.

## Supplementary information

**Additional file 1.** Supplementary materials: methods, figures and tables.

## Data Availability

Raw hMeDIP-seq data and MACS2 peaks supporting the conclusions of this article have been deposited in NCBI’s Gene Expression Omnibus and can be accessed with accession number GSE144034. RNA-seq data is available at GSE74956.

## Abbreviations

5mC: 5-Methylcytosine
5hmC: 5-Hydroxymethylcytosine
BMP: Bone morphogenic protein
ANOVA: Analysis of variance
DhMRs: Differentially hydroxymethylated regions
EMT: Epithelial to mesenchymal transition
FDR: False discovery rate
GO: Gene ontology
hMeDIP-Seq: Hydroxymethylated DNA immunoprecipitation sequencing
HNSCC: Head and neck squamous cell carcinoma
HPV: Human papillomavirus
IMU: Immune response
KRT: Keratinization
NHEK: Normal human epidermal keratinocyte
OR: Odds ratio
PC: Principle component
PCA: Principle component analysis
SCC: Squamous cell carcinoma
TCGA: The Cancer Genome Atlas
TET: Ten-eleven translocation
TES: Transcription end site
TGFB: Transforming growth factor beta
TSS: Transcription start site
SVD: Singular value decomposition
