# Supplementary material for: 5-Hydroxymethylation highlights the heterogeneity in keratinization and cell junctions in head and neck cancers
Source: Clin Epigenetics. 2020 Nov 17;12:175. doi: 10.1186/s13148-020-00965-8 (PMC7672859; doi:10.1186/s13148-020-00965-8)
Supplement: Supplementary file 1 — Additional file 1. Supplementary materials: methods, figures and tables. [file 13148_2020_965_MOESM1_ESM.docx]

**Additional File 1**

**Supplementary Materials: Methods, Figures and Tables**

For manuscript: 5-hydroxymethylation highlights the heterogeneity in keratinization and cell junctions in head and neck cancers

Siyu Liu, Marcell Costa de Medeiros, Evan M. Fernandez, Katie R. Zarins, Raymond G. Cavalcante, Tingting Qin, Gregory T. Wolf, Maria E. Figueroa, Nisha J. D’Silva, Laura S. Rozek, Maureen A. Sartor

**Supplementary Methods**

**Tumor tissue acquisition**

With written informed consents from patients and approval from University of Michigan Institutional Review Board, pretreated tumor tissues were collected for these patients, stored and flash frozen in liquid nitrogen at -80C. For each sample, H&E slides were prepared before passing the assessment criteria of at least 70% cellularity and less than 10% necrosis. Surface scrapings were then extracted from the tissue region where at least 70% tumor cellularity was confirmed while remaining frozen, followed by processing with Qiagen AllPrep DNA/RNA/Protein Mini Kit (Valencia, CA, USA) as per manufacturer protocol.

**Determination of important genes in differential 5hmC analysis**

The genes that are important in differential 5hmC analysis are those with highest number of DhMRs, those with the most significant DhMRs, those with DhMRs on promoters, and those that are related to cancer, especially HNSCC. A list of cancer-related and HNSCC-related genes were downloaded from COSMIC(1).

**Generation of custom enhancer definitions**

We defined a consensus set of human distal enhancers (>5kb from a TSS) from a combination of sources for enhancer locations and links to their target genes. The definition was based on enhancers from DNase hypersensitive sites (DHSs) from 125 cell types processed by ENCODE(2), distal and non-promoter DHS within 500kb of the correlated promoter DHSs from 32 cell types(2) and FANTOM5(3). All enhancer regions were extended up to 1kb around their midpoint. To identify target genes, we used enhancer and gene interactions (<5kb from TSS) identified by ChIA-PET2(4) in 13 ChIA-PET datasets(5,6) from 6 cell lines.

**RNA-seq analyses and association with 5hmC**

RNA-seq data from the same 36 HNSCC samples as for hMeDIP-seq was downloaded from GEO (accession number GSE74956)(7). The raw sequences were aligned to hg19 using Tophat2 v2.0.11(8), and gene expression count data were generated with HTSeq(9). Differential expression levels were compared both by HPV status and by HPV(+) subtypes using edgeR-robust, as described in (10). HPV status was previously determined by viral gene expression; briefly, samples with more than 500 read pairs aligned to any high-risk HPV genome were classified as HPV(+)(7). When comparing gene expression with 5hmC levels, for each identified DhMR by HPV-status, we calculated the log fold change (logFC) of 5hmC level as log HPV(+) / HPV(-), so that one gene corresponds to one gene expression logFC value and one or multiple 5hmC logFC values. The odds ratio (OR) of 5hmC logFC and gene expression logFC was calculated with a standard 2x2 contingency table containing the number of sites in each quadrant of **Figure 3A**.

**Gene set enrichment testing on hmeDIP-seq and RNA-seq data**

RNA-Enrich(11) was used for enrichment analysis on RNA-seq results, with the directional analysis option so that enrichment of gene sets in HPV(+) and HPV(-) samples could be distinguished.Gene Ontology (GO) term and pathway enrichment analysis for 5hmC peaks was conducted using the gene set enrichment program for genomic regions, ChIP-Enrich(12). The enrichment analysis on DhMR peaks was performed using the nearest TSS locus definition in ChIP-Enrich, and significant pathways were marked with an FDR < 0.1(12). Only “enriched” GO terms were selected for follow-up analyses. Broad GO terms with more than 1,500 genes were removed to keep the results more specific.

**Keratinocyte enhancer regions download and analysis**

ChromHMM(13) tracks of Primary Normal Human Epidermal Keratinocytes (NHEK) were downloaded from the UCSC Genome Browser, and the subset of regions overlapping between the ChromHMM track (strong enhancer, weak enhancer, active promoter and weak promoter regions only) and DhMRs by HPV status were identified using the *GenomicRanges* Bioconductor package. NHEK super-enhancers were defined using ROSE (Rank Ordering of Super-Enhancers)(14,15), and were downloaded from DbSUPER(16). NHEK ChIP-seq data for 9 histone marks (H3K27ac, H3K37me3, H3K36me3, H3K4me1, H3K4me2, H3K4m3, H3K9ac, H3K9me1, H4K20me1) were downloaded from ENCODE. The overall 5hmC levels over active enhancers and weak enhancers were calculated using MACS2 peaks in HOMER(17).

**Experimental Validation with HPV(+) and HPV(-) cell lines**

Cell lines

HPV negative (UM-SCC-1 and UM-SCC-29) and positive (UM-SCC-47, SCC-090 and

SCC-154) cells lines were plated to generate conditioned media, as described in (18). After conditioned media collection, cells were counted and divided equally for RNA and immunoblot assays.

Gelatin zymography

After normalized by cell number with blank media (1.8 x10^6^ cells/ml), conditioned media was concentrated in Centrifugal Filter Units (Millipore). Gelatin enzyme activity for MMP2 was evaluated by zymography(19).

RT-qPCR

Total RNA was isolated from cells lysed with Qiazol using miRNAeasy kit (Qiagen, Valencia, CA) and cDNA was synthesized using SuperScript II (Invitrogen, Carlsbad, CA). qPCR reaction was performed with the primers targeting MMP2 (FP - AGC GAG TGG ATG CCG CCT TTA A; RP - CAT TCC AGG CAT CTG CGA TGA G) and GAPDH using SYBR Green PCR Master Mix (#4309155, Applied Biosystem) on a StepOne plus Real-time PCR machine. Data were analyzed by the relative quantification method with normalization to GAPDH. Each sample was run in duplicate.

Immunoblot analysis

Immunoblots were performed as described in (20). Primary antibodies were anti-MMP2 (1:1000, #87809T, Cell Signaling Technology, Boston, MA) and anti-Actin (1:2000, #612656) (BD Biosciences, Franklin Lakes, NJ). Secondary antibodies were horseradish peroxidase (HRP)-conjugated goat anti-rabbit IgG or goat anti-mouse IgG (Jackson Immuno Research Laboratories, West Grove, PA). Immunoreactive proteins were visualized by SuperSignal™ West Pico Chemiluminescent Substrate. Signal intensity was quantified using ImageJ and expressed as densitometric in **Figure 6B**.

**Supplemental References**

1. Tate JG, Bamford S, Jubb HC, Sondka Z, Beare DM, Bindal N, et al. COSMIC: The Catalogue Of Somatic Mutations In Cancer. Nucleic Acids Res. 2019;

2. Thurman RE, Rynes E, Humbert R, Vierstra J, Maurano MT, Haugen E, et al. The accessible chromatin landscape of the human genome. Nature. 2012;489:75–82.

3. Lizio M, Harshbarger J, Shimoji H, Severin J, Kasukawa T, Sahin S, et al. Gateways to the FANTOM5 promoter level mammalian expression atlas. Genome Biol. 2015;16.

4. Li G, Chen Y, Snyder MP, Zhang MQ. ChIA-PET2: A versatile and flexible pipeline for ChIA-PET data analysis. Nucleic Acids Res. 2017;

5. Dowen JM, Fan ZP, Hnisz D, Ren G, Abraham BJ, Zhang LN, et al. Control of cell identity genes occurs in insulated neighborhoods in mammalian chromosomes. Cell. 2014;

6. Tang Z, Luo OJ, Li X, Zheng M, Zhu JJ, Szalaj P, et al. CTCF-Mediated Human 3D Genome Architecture Reveals Chromatin Topology for Transcription. Cell. 2015;

7. Zhang Y, Koneva LA, Virani S, Arthur AE, Virani A, Hall PB, et al. Subtypes of HPV-positive head and neck cancers are associated with HPV characteristics, copy number alterations, PIK3CA mutation, and pathway signatures. Clin Cancer Res. 2016;22:4735–45.

8. Kim D, Pertea G, Trapnell C, Pimentel H, Kelley R, Salzberg SL. TopHat2: Accurate alignment of transcriptomes in the presence of insertions, deletions and gene fusions. Genome Biol. 2013;14.

9. Anders S, Pyl PT, Huber W. HTSeq-A Python framework to work with high-throughput sequencing data. Bioinformatics. 2015;31:166–9.

10. Robinson MD, McCarthy DJ, Smyth GK. edgeR: A Bioconductor package for differential expression analysis of digital gene expression data. Bioinformatics. 2009;

11. Lee C, Patil S, Sartor MA. RNA-Enrich: A cut-off free functional enrichment testing method for RNA-seq with improved detection power. Bioinformatics. 2016;32:1100–2.

12. Welch RP, Lee C, Imbriano PM, Patil S, Weymouth TE, Smith RA, et al. ChIP-Enrich: Gene set enrichment testing for ChIP-seq data. Nucleic Acids Res. 2014;42.

13. Ernst J, Kellis M. ChromHMM: Automating chromatin-state discovery and characterization. Nat. Methods. 2012. page 215–6.

14. Whyte WA, Orlando DA, Hnisz D, Abraham BJ, Lin CY, Kagey MH, et al. Master transcription factors and mediator establish super-enhancers at key cell identity genes. Cell. 2013;

15. Lovén J, Hoke HA, Lin CY, Lau A, Orlando DA, Vakoc CR, et al. Selective inhibition of tumor oncogenes by disruption of super-enhancers. Cell. 2013;

16. Khan A, Zhang X. DbSUPER: A database of Super-enhancers in mouse and human genome. Nucleic Acids Res. 2016;

17. Heinz S, Benner C, Spann N, Bertolino E, Lin YC, Laslo P, et al. Simple Combinations of Lineage-Determining Transcription Factors Prime cis-Regulatory Elements Required for Macrophage and B Cell Identities. Mol Cell. 2010;38:576–89.

18. Henson BS, Neubig RR, Jang I, Ogawa T, Zhang Z, Carey TE, et al. Galanin receptor 1 has anti-proliferative effects in oral squamous cell carcinoma. J Biol Chem. 2005;

19. Mitra RS, Goto M, Lee JS, Maldonado D, Taylor JMG, Pan Q, et al. Rap1GAP promotes invasion via induction of matrix metalloproteinase 9 secretion, which is associated with poor survival in low N-stage squamous cell carcinoma. Cancer Res. 2008;

20. Mitra RS, Zhang Z, Henson BS, Kurnit DM, Carey TE, D’Silva NJ. Rap1A and rap1B ras-family proteins are prominently expressed in the nucleus of squamous carcinomas: Nuclear translocation of GTP-bound active form. Oncogene. 2003;

**Supplementary Figures**

**Figure S1: Features of hyper-5hmC peaks in HPV(+) and HPV(-) tumors.** (A) Range of MACS2 peak number for HPV(+) and HPV(-) samples respectively. (B) Range of differential 5hmC peak lengths for HPV(+) and HPV(-) samples respectively, with HPV(-) peaks slightly longer than HPV(+) ones.

**Figure S2: Important tumor suppressor and cell adhesion genes were found with hyper-5hmC in HPV(+) and HPV(-) HNSCCs respectively.** (A) CDKN2A bam signal by sample showed that the coverage depth for HPV(+) tumors is generally higher than HPV(-) tumors, especially at the 5kb intron region. A total of 10 HPV(+) DhMRs were annotated to CDKN2A, and MACS2 signal values these peaks were significantly higher in HPV(+) samples than those in HPV(-) samples. 0 signal value means there is no MACS2 peak detected. (B) A total of 83 HPV(+) DhMRs were annotated to CDH13, and MACS2 signal values these peaks were significantly higher in HPV(-) samples than those in HPV(+) samples.

**Figure S3: Significantly enriched pathways in hyper-5hmC peaks of HPV(+) and HPV(-) tumors.** Bubble plot showing all significantly enriched (FDR < 0.1) GO terms in hyper-5hmC peaks of HPV(+) tumors and top 20 significantly enriched GO terms in those of HPV(-) tumors. The size of the dot indicates the number of genes in each GO term, and the color denotes the significance (red: more significant; blue: less significant).

**
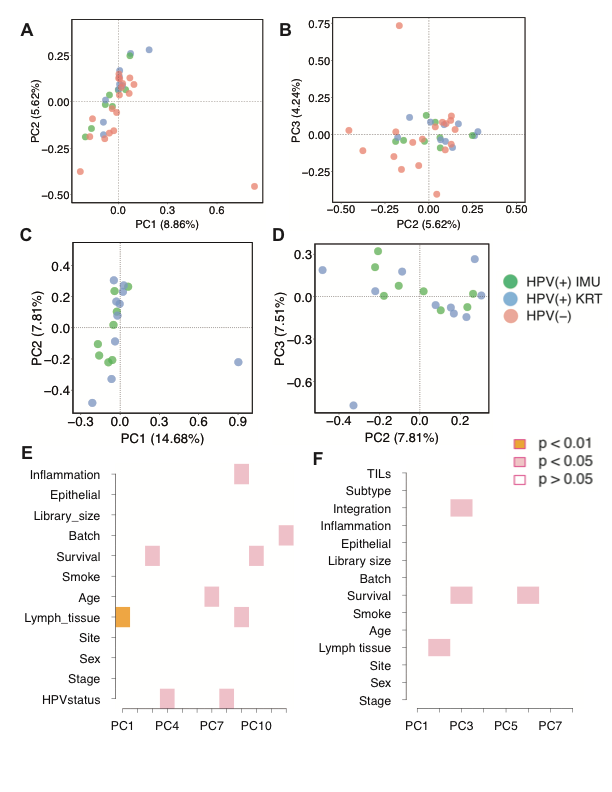
**

**Figure S4: Principle component analysis did not show any clear separation between the HPV(+) IMU subtypes and the rest for 5hmC level at gene body regions.** (A-D) Neither PC1 vs PC2 nor PC2 vs PC3 of 5hmC level on gene bodies showed any separation based on HPV status or HPV(+) subtype. (E-F) The SVD analysis on gene body regions demonstrated several relevant clinical variables, such as survival, lymphocyte tissue and HPV virus integration, which significantly correlated with each principle component in 36 tumor and 18 HPV(+) samples respectively.

**Figure S5: Features of hyper-5hmC peaks in HPV(+) IMU and HPV(+) KRT subtypes.** (A) The normalized global 5hmC distribution pattern over gene bodies in HPV(+) IMU, HPV(+) KRT and HPV(-) samples. (B) Venn diagram showing the number of overlapped peaks between hyper-5hmC peaks based on HPV status and HPV(+) subtype. The table on the right further splits the overlapped peaks into hyper-5hmC in HPV(+) / HPV(-) and HPV(+) IMU / HPV(+) KRT. (C) The distribution of hyper-5hmC peaks from HPV(+) IMU and HPV(+) KRT subtypes, where first column represents the combination of both IMU and KRT peaks. There are slightly more DhMRs annotated to 1-5kb TSS and exons in KRT than IMU. (D) Enrichment analysis results for gene expression vs hydroxymethylation based on HPV(+) subtype. Each dot represents one GO term, and the color denotes the significance (yellow: significant; blue: not significant). Signed p-values are defined as >0 to indicate up-regulation in HPV(+) IMU subtype or hyper-hydroxymethylation in HPV(+) IMU subtype, and <0 to indicate upregulation in HPV(+) KRT subtype or hyper-hydroxymethylation in HPV(+) KRT subtype.

**Figure S6: Significantly enriched pathways in hyper-5hmC peaks of HPV(+) IMU and HPV(+) KRT subtypes.** Bubble plot showing top 20 significantly enriched (FDR < 0.1) GO terms in hyper-5hmC peaks of HPV(+) IMU samples and HPV(+) KRT samples respectively. The size of the dot indicates the number of genes in each GO term, and the color denotes the significance (red: more significant; blue: less significant).

**Figure S7: Correlation between gene expression and 5hmC.** Scatterplot showing the positive correlation between gene expression and hydroxymethylation at enhancer, propmoter, and gene body regions separately (Pearson correlation coefficient = 0.49, 0.39 and 0.53 respectively). The top half represents genes that are significantly up-regulated in HPV(+) tumors, and the right half represents genes that have hyper-5hmC peaks in HPV(+) tumors.

**Figure S8: Network analysis using shortest paths in Metacore.** The results showed the center nodes in (A) HPV(+) and (B) HPV(-) samples respectively.

**Figure S9: Genome-wide distribution of over enhancers and promoters showed different patterns.** Global levels of 5hmC over NHEK active enhancers and weak enhancers in HPV(+) IMU, HPV(+) KRT and HPV(-) samples.

|  | **Total** | **HPV(+)** | **HPV(-)** |
| --- | --- | --- | --- |
|  | 36 | 18 | 18 |
| **Age** |  |  |  |
| Median (std) |  | 60.1 (7.3) | 58.4 (10.2) |
| **Gender** |  |  |  |
| Male | 26 | 17 | 9 |
| Female | 10 | 1 | 9 |
| **HPV type** |  |  |  |
| HPV16 | 14 | 14 |  |
| HPV18 | 1 | 1 |  |
| HPV33 **Anatomical Site** | 1 | 1 |  |
| Oropharynx Oral Cavity Larynx **Tumor Stage** | 20  14  2 | *17 (94%)*  *1 (10%)*  0 | *3 (17%)*  *13 (72%)*  2 *(11%)* |
| I-II | 5 | 1 | *4* |
| III | 3 | 2 | *1* |
| IV | 28 | 15 | *13* |
| **T stage** |  |  |  |
| T1-T2 | 14 | 8 | *6* |
| T3-T4 | 22 | 10 | *12* |
| **N stage** |  |  |  |
| N0 | 10 | 4 | *6* |
| N1 | 2 | 1 | *1* |
| N2 | 17 | 10 | *7* |
| N3 | 7 | 3 | *4* |
| **Smoking** |  |  |  |
| Never | 7 | *4 (22%)* | *3 (17%)* |
| Former | 23 | *11 (61%)* | *12 (66%)* |
| Current | 6 | *3 (17%)* | *3 (17%)* |

**Table S1**: Demographic information for the University of Michigan HNSCC patient cohort. Note that 2 larynx samples were originally classified as oropharynx, but later reclassified to larynx.

| Genes with highest number of DhMRs (>10) | CSMD1, XKR4, MTAP, TMEM178B, EPHA6, ADARB2, ROBO1, LINGO2, MIR924HG, CDKN2A |
| --- | --- |
| Genes with most significant DhMRs (FDR < 10^-7^) | CDKN2A, MTAP, C5orf64, NAALADL2, RPH3A, SLC7A9, AMPH, PPP2R2B, PCLO |
| Genes with DhMRs on promoters | CDKN2A, MOV10, SEMA6A, ZSWIM2, PCLO, CLDN10, CSMD1, UBE2H, ACOT7, COL21A1, DOCK9, etc. |
| Cancer-related genes with significant DhMRs | CDKN2A, BTG1, ANGPT2, ZBTB7C, DCC, CEACAM1, CDKN2B, TCF7L2, TIAM1, OCA2, TP63, KIT |
| HNSCC-related genes with significant DhMRs | CDKN2A, CSMD1, ZBTB7C, SMC1B, TP63 |

**Table S2A**: Important genes found with HPV(+) DhMRs

| Genes with highest number of DhMRs (>40) | CDH13, SYN3, PRKCA, EML1, FOXN3, TTC7B, EML1, RCOR1, TRIO, CACNA1C, CMIP, GAS7 |
| --- | --- |
| Genes with most significant DhMRs (FDR < 10^-9^) | SMARCC1, STRN3, AP4S1, UNC13A, CCDC102B, KCNIP4, GALNT9, GRM1, SLC6A5, MYH16, RNF185, CPNE4 |
| Genes with most significant DhMRs on promoters | CMIP, PAX8-AS1, SEPT4, PAPLN, IL32, PPFIA1, VAC14, FKBP15, SMAD3, ABCC2, BCAR1, MMP2, etc. |
| Cancer-related genes with significant DhMRs | ZFHX3, TIMP2, RUNX1, BCAR1, DAPK1, GLI2, WWOX, FERMT2, RAD51B, ABCC2, TIMP3 |
| HNSCC-related genes with DhMRs on promoters | MACF1, FANCA, NADSYN1, LINC00111, RCN1 |

**Table S2B**: Important genes found with HPV(-) DhMRs

| Genes with highest number of DhMRs (>100) | SLIT3, AUTS2, NFIB, FHOD3, TENM3, ETV6, ADARB2, SDK1, SYNPO2, DHRSX |
| --- | --- |
| Genes with most significant DhMRs (FDR < 10^-16^) | MYBPC1, PIP5K1B, IL31RA, FAF2, NFIB, TP53TG5, COL23A1, SLC25A21, B4GALT6, PTPRA |
| Genes with most significant DhMRs on promoters | TP53TG5, PTPN3, LOC158434, B4GALNT2, MICALL1, ADRA1B, CCDC8, RNF150, SPOCK2 |
| Cancer-related genes with most DhMRs | BCL2, PDZD2, RUNX1, OCA2, WWOX, ERC1, MGMT, TP73, FGFR2, TCF7L2, TG, TSPAN8 |
| HNSCC-related genes with most DhMRs | CSMD1, CTIF, TEAD1, ZBTB7C, KLF12, MACF1, FANCC, RAD51B, PBX1, BCAS3, SYNJ2 |

**Table S3A**: Important genes found with HPV(+) IMU DhMRs

| Genes with highest number of DhMRs (>5) | CMIP, ANKRD11, TANGO6, GPT2, CDH13, GAN, GLG1, CNST, CTBP2, PHLDB2, TOM1L2 |
| --- | --- |
| Genes with most significant DhMRs (FDR < 10^-7^) | MAMDC2, PCSK5, NYAP2, KCNA2, ZFR, SMARCC1, RSPO1, MYH16, SYT2 |
| Genes with most significant DhMRs on promoters | CHD7, LPAR6, ZNF365, LEP, KCNK6, ARHGAP23, LOC93429, ITFG1, SNX5, SNORD17 |
| Cancer-related genes with most DhMRs | CDH13, TP63, ABCC5, CCDC6, CDH1, EXT1, TPD52L1, WWOX, BCAR1, KANK1, LRRC4, NQO1, PXN |
| HNSCC-related genes with DhMRs | PHLDB2, TP63, PLCXD2, GPD2, MACF1, ARAP3, ARHGEF3, BRIP1, ITSN2, MAML3, TBC1D1, TBC1D9B, TGFBR2 |

**Table S3B**: Important genes found with HPV(+) KRT DhMRs

| GO term names | FDR in ChIP-Enrich | FDR in RNA-Enrich |
| --- | --- | --- |
| cellular component morphogenesis | 7.52E-06 | 2.22E-02 |
| cell morphogenesis | 2.34E-05 | 4.72E-02 |
| cytoskeletal protein binding | 8.36E-05 | 2.90E-02 |
| cell projection organization | 1.85E-04 | 1.21E-02 |
| cell projection morphogenesis | 1.85E-04 | 1.76E-02 |
| actin binding | 1.15E-03 | 7.51E-03 |
| adherens junction | 7.32E-03 | 3.12E-08 |
| chemotaxis | 1.23E-03 | 2.35E-02 |
| neuron development | 1.40E-03 | 1.83E-02 |
| actin filament-based process | 1.40E-03 | 1.64E-02 |
| actin cytoskeleton organization | 1.40E-03 | 4.31E-02 |
| regulation of anatomical structure morphogenesis | 1.50E-03 | 3.86E-02 |
| cell leading edge | 7.32E-03 | 8.40E-04 |
| cell-substrate junction | 7.32E-03 | 4.54E-09 |
| focal adhesion | 7.32E-03 | 2.69E-09 |
| ruffle | 7.32E-03 | 6.73E-06 |
| anchoring junction | 7.32E-03 | 1.80E-08 |
| cell-substrate adherens junction | 8.41E-03 | 2.69E-09 |
| negative regulation of response to stimulus | 5.53E-03 | 1.72E-02 |
| cell projection | 1.53E-02 | 8.32E-05 |
| cytoplasmic vesicle | 1.81E-02 | 1.91E-03 |
| vesicle | 2.07E-02 | 6.33E-13 |
| endoplasmic reticulum lumen | 2.07E-02 | 1.45E-03 |
| integrin binding | 2.48E-02 | 1.84E-02 |
| membrane organization | 4.33E-02 | 3.98E-02 |

**Table S4:** Gene set enrichment results revealed pathways that are enriched in both HPV(-) hyper-hydroxymethylation and HPV(-) gene up-regulation.

| GO term names | FDR in ChIP-Enrich | FDR in RNA-Enrich |
| --- | --- | --- |
| cellular component morphogenesis | 7.52E-06 | 2.22E-02 |
| cell morphogenesis | 2.34E-05 | 4.72E-02 |
| cytoskeletal protein binding | 8.36E-05 | 2.90E-02 |
| cell projection organization | 1.85E-04 | 1.21E-02 |
| cell projection morphogenesis | 1.85E-04 | 1.76E-02 |
| actin binding | 1.15E-03 | 7.51E-03 |
| adherens junction | 7.32E-03 | 3.12E-08 |
| chemotaxis | 1.23E-03 | 2.35E-02 |
| neuron development | 1.40E-03 | 1.83E-02 |
| actin filament-based process | 1.40E-03 | 1.64E-02 |
| actin cytoskeleton organization | 1.40E-03 | 4.31E-02 |
| regulation of anatomical structure morphogenesis | 1.50E-03 | 3.86E-02 |
| cell leading edge | 7.32E-03 | 8.40E-04 |
| cell-substrate junction | 7.32E-03 | 4.54E-09 |
| focal adhesion | 7.32E-03 | 2.69E-09 |
| ruffle | 7.32E-03 | 6.73E-06 |
| anchoring junction | 7.32E-03 | 1.80E-08 |
| cell-substrate adherens junction | 8.41E-03 | 2.69E-09 |
| negative regulation of response to stimulus | 5.53E-03 | 1.72E-02 |
| cell projection | 1.53E-02 | 8.32E-05 |
| cytoplasmic vesicle | 1.81E-02 | 1.91E-03 |
| vesicle | 2.07E-02 | 6.33E-13 |
| endoplasmic reticulum lumen | 2.07E-02 | 1.45E-03 |
| integrin binding | 2.48E-02 | 1.84E-02 |
| membrane organization | 4.33E-02 | 3.98E-02 |
| cellular component morphogenesis | 7.52E-06 | 2.22E-02 |
| cell morphogenesis | 2.34E-05 | 4.72E-02 |
| cytoskeletal protein binding | 8.36E-05 | 2.90E-02 |
| cell projection organization | 1.85E-04 | 1.21E-02 |
| cell projection morphogenesis | 1.85E-04 | 1.76E-02 |
| actin binding | 1.15E-03 | 7.51E-03 |
| adherens junction | 7.32E-03 | 3.12E-08 |
| chemotaxis | 1.23E-03 | 2.35E-02 |
| neuron development | 1.40E-03 | 1.83E-02 |
| actin filament-based process | 1.40E-03 | 1.64E-02 |

**Table S5A**: Gene set enrichment results revealed pathways that are enriched in both HPV(+) IMU hyper-hydroxymethylation and HPV(+) IMU gene up-regulation.

| GO term names | FDR in ChIP-Enrich | FDR in RNA-Enrich |
| --- | --- | --- |
| cadherin binding | 3.97E-07 | 3.46E-05 |
| cell-cell junction | 2.11E-04 | 2.20E-05 |
| cell adhesion molecule binding | 7.32E-04 | 1.56E-02 |
| keratinocyte differentiation | 3.76E-03 | 3.53E-08 |
| cornified envelope | 2.29E-03 | 4.08E-10 |
| cadherin binding involved in cell-cell adhesion | 4.60E-03 | 3.46E-05 |
| protein binding involved in cell-cell adhesion | 5.43E-03 | 3.46E-05 |
| lateral plasma membrane | 1.80E-02 | 1.52E-02 |
| protein binding involved in cell adhesion | 2.08E-02 | 3.46E-05 |
| cell-cell adherens junction | 2.40E-02 | 1.03E-05 |
| epidermal cell differentiation | 4.17E-02 | 7.78E-07 |

**Table S5B:** Gene set enrichment results revealed pathways that are enriched in both HPV(+) KRT hyper-hydroxymethylation and HPV(+) KRT gene up-regulation.
